# Route Optimization Tool (RoOT) for distribution of vaccines and health products

**DOI:** 10.12688/gatesopenres.13219.1

**Published:** 2021-03-01

**Authors:** Zelda B. Zabinsky, Mariam Zameer, Larissa P.G. Petroianu, Mamiza M. Muteia, Aida L. Coelho

**Affiliations:** 1 Dept. of Industrial & Systems Engineering, University of Washington, Seattle, Seattle, WA, 98195, USA; 2 VillageReach, Seattle, WA, USA; 3 VillageReach, Maputo, Mozambique

**Keywords:** Supply chain distribution, vaccines, cold chain, medical supplies, health products, vehicle routing, computational modeling, optimization, direct delivery, system design, risk, transit time, COVID-19, route optimization, system design, light-touch tools, optimized routing

## Abstract

Delivery of health products from provinces or districts to health facilities, including temperature-sensitive vaccines, is one of the most effective interventions to ensure availability of supplies and save lives in low- and middle-income countries. Currently, routes are hand drawn by logisticians that are adjusted based on vehicle availability and quantity of products. Easy-to-use supply chain tools are needed that planners can use in real-time to create or adjust routes for available vehicles and road conditions. Efficient and optimized distribution is even more critical with the COVID-19 vaccine distribution.

We develop a Route Optimization Tool (RoOT) using a variant of a Vehicle Routing and Scheduling Algorithm (VeRSA) that is coded in Python, but reads and writes Excel files to make data input and using outputs easier. The tool takes into account cold chain distribution, is easy-to-use, and provides routes quickly within two minutes. RoOT can be used for routine operations or in emergency situations, such as delivery of new COVID-19 vaccine. The tool has a user-centric design with easy dropdown menus and the ability to optimize on time, risk, or combination of both.

Training of logisticians in Mozambique indicate that RoOT is easy to use and provides a tool to improve planning and efficient distribution of health products, especially vaccines. We illustrate using RoOT in an emergency situation, such as a cyclone.

RoOT is an open-source tool for optimal routing of health products. It provides optimized routes faster than most commercial software, and is tailored to meet the needs of government stakeholders. Currently, RoOT does not allow multi-day routes, and is designed for trips that can be completed within twenty-four hours. Areas for future development include multi-day routing and integration with mapping software to facilitate distance calculations and visualization of routes.

## Introduction

Vaccines save millions of lives every year and save billions of dollars by reducing health care costs and preventing lost productivity ^1^, yielding an estimated 10- to 25-fold return on investment ^2^. Despite their effectiveness, global vaccination coverage has plateaued at 85% since 2010 and, in 2017, an estimated 20 million children lacked access to routine vaccination services—approximately 60% of whom lived in resource-constrained countries ^3^. One reason for stagnating coverage rates is inefficiencies in the immunization supply chain, which is increasingly challenged by population growth, new vaccine introductions, currency and policy fluctuations, and the introduction of new technologies and supply chain practices ^4^. This is even more critical today, with the COVID-19 pandemic and the efficient distribution of the COVID-19 vaccine. Many governments and stakeholders looked to a COVID-19 vaccine when the pandemic hit. Now that the vaccine doses are arriving in low and middle income countries, it is important to plan for the efficient and optimized distribution.

Direct delivery of health products to health facilities, by districts or provinces, is one of the most effective interventions to improve product availability and reduce risk to vaccines. This is not only cost effective but gives health workers more time to provide services, and improve coverage, that would instead be spent traveling to pick up health products. Based on VillageReach’s experience leading supply chain system design efforts using modeling tools in the Democratic Republic of the Congo, Mozambique, Pakistan, and Zambia ^5– 7^, direct delivery reduces stockouts, and contributes to improving equity. Additionally, health products, especially vaccines, are most at risk of temperature excursions during transit ^8– 10^; having products packed well and delivered efficiently to health facilities, reduces risk to products and maintains their potency. This is important for all vaccines, but especially so for COVID-19 vaccines due to limited supply, high demand, and the special ultra cold chain requirements for some vaccines.

For direct delivery, one of the most important decisions made by governments is effective routing that delivers products safely and quickly. Currently, routes are hand drawn by logisticians, and are not optimized for transit time or road conditions to improve availability and maintain vaccine potency. There is no tool available to allow logisticians to adjust plans quickly when the vehicle availability or situation changes, such as when a vehicle breaks down or a road is impassable due to flooding. Direct delivery distribution depends on vehicle availability, which often varies month to month, as well as the capacity of the vehicle for delivery. There are currently no supply chain tools and methods that logisticians can use in real-time to easily decide which vehicles to use for specific routes, and to quickly calculate if they can carry the required product volumes.

In 2019, Mozambique experienced Cyclone Idai, which devastated many health facilities across the country. Supply chains were disrupted leading to stockouts and unavailability of health products. Hence, an approach was needed to adjust supply chains to account for the road infrastructure that was destroyed, making some areas completely inaccessible, as well as changes to storage locations as some facilities were damaged. Additionally, make-shift facilities were set up in other areas to serve communities. Even for health facilities whose physical infrastructure was not impacted, their ability to maintain cold storage for vaccines was affected with disruptions in electricity in addition to the increasing numbers of people they were serving. This called for a shift in design of the supply chain system, and highlighted the need for a quick and easy way for logisticians to distribute health products where they were needed the most.

Based on our experience, we recognize the need for a new tool that can be used by governments and organizations who do not have the time, resources, ability, willingness, or political capital to conduct extensive optimization or simulation modeling that can sometimes take days to run, but will benefit from a fast and easy-to-use tool that provides results in minutes. Hence, we designed the Route Optimization Tool (RoOT), keeping in mind delivery of health products in routine and emergency settings, as well as the time and skills of government users.

### Landscaping of existing methods and tool

Before developing RoOT, we did a literature review of the existing tools and methods available for optimizing distribution of health products based on minimizing risk of spoilage of health products. While there is extensive literature on the importance of transportation for health supply chains, infrastructure risks and financial systems are rarely addressed, and these are usually responsible for most of the network disruptions ^11^. Often, the routing decision is based on the shortest distance, as it presumably reduces cost and leads to faster delivery. However, other risk factors, such as road failure from flooding, road sink, or bridge collapse, could make a recommended route infeasible ^12, 13^.

One option for optimizing routes is to incorporate the risk of road and infrastructure failures into the primary objective, instead of solely minimizing transit time or distance. Penalty parameters can be used as a way to incorporate the probability of road failure into an objective function, thus enabling the optimal routes to avoid unreliable roads, as in Hamedi *et al.* ^13^ Studies show that using a minimum risk approach identifies routes that avoid critical roads and thus decrease risk ^14^.

While analyzing risks for routing delivery of health products is not common ^11^, risk is often used as the main objective when transporting hazardous materials ^15, 16^. Risk of spilling hazardous materials is addressed with the probability of accidents due to speed, road conditions, and busy intersections ^17^. Accident rates are also assessed due to the time of day, weather conditions, and type of road ^16, 18^. Using risk minimization, routes that avoid these dangers are preferred, even if there is an increase in travel time, as they ensure safe transport and delivery of materials.

In redesigning the supply chain for distribution of health products including vaccines and temperature sensitive products, we investigated different optimization tools that are available, including tools that perform inventory optimization, network optimization (minimize cost or distance) or route optimization. Since one of the main contributors to waste and spoilage of vaccines and temperature sensitive products is not maintaining effective cold chain during transport, we sought optimization tools that capture risk as well as transit time ^19^. However, most decision support systems for logistics focus on inventory control and network optimization, and are not easily adapted to balance risk with transit time for route optimization ^20^.

During the landscaping analysis, we identified 18 vehicle routing software packages and 15 supply chain software packages that could be used in our context ^21^. Most of the routing software used in commercial logistics focuses on distribution efficiency to minimize cost and time, and does not explicitly include risk as an objective. A user cannot easily modify these tools to tailor the objective functions and constraints for health products. Additionally, commercially available tools are costly, require special installation and training, may need to run for several hours to provide a solution, and are not easy-to-use or “light”.

We narrowed down the 15 identified supply chain logistics software to two that had capabilities for optimizing routes: LLamasoft - Supply Chain Guru® Cloud-Based Supply Chain Design Software, and Global Logistic Competence (GLC) ^22, 23^. Our prior experience with using these tools was that they were complex to use, costly, and required advanced skills. Also, they require detailed data that is often not readily available in low- and middle-income countries (LMICs), such as for Mozambique.

The landscaping analysis identifies a clear need for a light-tool that can be used easily by a variety of stakeholders, without the need for advanced user skills or significant financial investment. There is also a need to tailor a software tool to represent considerations of risk of spoilage of health products.

## Methods

### Model description

The optimization model in RoOT is a variation of a vehicle routing optimization problem ^24^ that is tailored to address the needs of a cold chain distribution of temperature sensitive health products (such as vaccines) and ambient temperature health products (such as syringes or medicines). RoOT is designed to be easy to use, and is based in Microsoft Excel since it is a software most users in governments and local organizations working on supply chains are familiar with. VillageReach’s experience in supporting government users in Mozambique was leveraged to ensure the optimization model could address common supply chain questions as summarized in Box 1. 

The input data for RoOT has seven sheets in Microsoft Excel:

1. Parameters: includes basic parameters and option to select optimization function
2. Products: list of all health products and temperature for storage
3. Center Capacities: cold and ambient storage capacities
4. Demand: quantities of health products needed at each facility
5. Vehicle: vehicles available for use, including condition
6. Distance Data: matrix of distances between facilities
7. Road Condition: matrix of road conditions between facilities.

RoOT creates an output file in Microsoft Excel that details routes for each available vehicle, with departure times from each facility on its route, the complete list of health products to be delivered with quantities, and the estimated transport cost. There are two sheets in the Excel output file:

1. Routes: detailed description of each route and associated vehicle used to complete the distribution, including times leaving each health facility, utilization of vehicle capacity on each route, and fuel and per diem costs, and
2. Health products: detailed description of the quantity of health products transported by each vehicle on each route and delivered to each health facility.

A complete description of the inputs and outputs is available in the user’s guide on GitHub ^25^.

**Box 1. Modeling support needed by government users**

RoOT is designed to meet the modeling needs of government users, based on questions that government stakeholders are typically interested in modeling:

- How does changing the resupply frequency, impact the quantity delivered at each distribution?
- What adjustments are needed when a new vaccine or health product is added to a distribution route?
- What vehicle is needed and routes adjusted if a new health facility is added to distribution route?
- What is the transport cost for each distribution route option?
- What changes in routes are needed if a road is unavailable or if the road condition changes (e.g., rain, flood, conflict or natural disaster)?
- What adjustments are needed when storage capacities change, either decreasing due to natural disaster or requirements of product (e.g. requiring ultra cold chain) or increasing with added cold chain when new refrigerators are added?
- How do I optimize distribution routes when a new vehicle is added or when a vehicle breaks down?
- How do I optimize distribution during an emergency or outbreak, or need for immediate distribution?

The optimization model in RoOT has two objectives and seven types of constraints, as summarized in Box 2. Our approach in RoOT is to allow the user to explore the trade-off between transit time and risk by providing two objective functions. The user can choose to minimize transit time or minimize risk by choosing penalties, and comparing the resulting routes. Alternately, users can also create an objective function by weighting transit time and risk to find a route that balances both objectives. The objective functions are described in more detail in the next section, *Multiple objectives*.

The constraints ensure that the routes can be practically implemented, such as only traveling on roads that are accessible, vehicles only carry supplies based on capacity, and supplies are provided based on demand at health facilities. A complete discussion is in the section *Constraints and assumptions*.

**Box 2. Route Optimization Tool (RoOT) objectives, constraints, and outputs**

Optimization Objectives:

- Minimize transit time
- Minimize risk penalty

Constraints:

- Maximum time spent for each route during a single day
- Vehicles start and return to the same facility
- Demand request from facilities is always met
- Each health facility is visited by exactly one vehicle
- All available vehicles are used as evenly as possible
- Supplies transported on each route do not exceed vehicle capacity for cold and ambient health products, by vehicle type
- All available roads can be used for distribution

Solution Outputs:

- Vehicle routes
- Time to depart each facility on route
- Quantity of health products delivered
- Cost (fuel and per diem) of routes

Based on user needs and the goal to have easy-to-use modeling tools, VillageReach and the University of Washington agreed on the requirements for the Route Optimization Tool (RoOT). The tool should:

- Be easy to use and easy to modify data by governments and technical partners and, hence, be based in Microsoft Excel.
- Be usable for routine operations, but also in emergency situations.
- Consider all health products, including those that need cold chain, such as vaccines that need to be kept at an ideal temperature range during transport.
- Consider the availability and reliability of vehicles.
- Consider the road conditions that may change seasonally (e.g. flooding) or in emergencies.
- Provide routes with departure times and quantities of health products for delivery.
- Minimize transit time and risk to vaccines.
- Calculate cost of routes.
- Execute quickly and provide results within minutes.

The steps we took to design an easy-to-use tool are described in the section *Usability*.

In addition to the tool being easy to use, it is also important that the tool provides a solution quickly, since most government users would not have the time to use a tool that would take hours to run. As described in more detail in the section *Computational performance*, we tested several available optimization algorithms and observed that it may take hours to produce a feasible solution, and even longer to determine an optimal solution. We decided to develop our own optimization algorithm that we could fine-tune to provide a solution to our optimization model quickly (see *Computational performance*).

### Multiple objectives

Efficient distribution is critical in supply chains to ensure the supplies reach facilities in time and that there are no stockouts. Further, vehicles are often used for multiple purposes, such as supervision, training, outreach, asides from deliveries. Hence, *minimize transit time* was chosen as the first parameter or objective function available to the user. Most vaccines are also often at risk of spoilage during transit; this probability is also reduced by minimizing transit time.

It was also important to *minimize the risk* of temperature excursion by using the best available roads and vehicles during transport. Most vaccines need to be stored between 2–8°C and exposure to temperatures outside this range results in vaccines losing potency. Hence, even if vaccines or other temperature-sensitive commodities reach the service delivery point, but are not potent, they are not effective. Since vaccines are also at increased risk for losing potency if a vehicle breaks down en route, vehicle condition impacts risk. In addition, if a vehicle gets stuck on a road due to pot-holes or water on the road, road condition also impacts risk. Hence, road and vehicle condition are associated with penalties and defined in the second objective in the optimization model. This is a critical consideration for all vaccines, but more so for the COVID-19 vaccine given the specialized temperature requirements, high demand, limited supply, and associated costs related to vaccine procurement.

RoOT allows users to balance transit time with risk by entering a weight for transit time, denoted *W _t_*, between 0 and 10, and then the weight for risk, denoted *W _p_*, which is calculated as *W _p_* = 10- *W _t_*. The objective in RoOT is:

$$\text{minimize}\;W_{t}\;\text{(}transit\;time\;of\;all\;routes\text{)}+W_{p}(risk\;for\;all\;routes).$$

If the user enters *W _t_* = 10, then the model will only optimize transit time, and if *W _t_* = 0 then the model will only optimize the risk from unreliable roads and vehicles, whereas any value in between will balance minimizing transit time and risk.

### Constraints and assumptions

The constraints of the optimization model in RoOT, as in Box 2, reflect the basic assumptions highlighted in Box 3.

**Figure 1.**
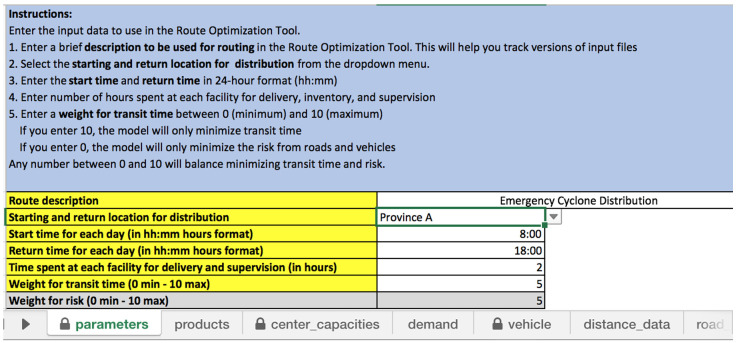
Spreadsheet for “parameters” input sheet, including the start and return location and times.

RoOT only considers single-day routes used in distributions. The corresponding constraint is a maximum time limit for a route set by the user, must be less than 24 hours. The user can choose to set this for 24 hours, representing 3 days of 8 hours each. Figure 1 illustrates the “parameter” input sheet where the start time and return time is specified.

The second assumption is that vehicles start and return to the same distribution warehouse. Although vehicles for distribution may be available at different locations, they would need to pick up health products at a specified distribution warehouse. Hence, it is a reasonable constraint and assumption that each route starts from and returns to the distribution warehouse.

**Box 3. RoOT model assumptions**

The assumptions in the model are:

1. Distribution routes are completed within one day, a maximum 24-hour time period.
2. Vehicles start and return to the same distribution warehouse.
3. There is sufficient supply of health products at the distribution warehouse to meet the requested demand.
4. Health facilities can properly store the entire quantity of health products requested.
5. Distribution routes use roads which vehicles can access (transport over water or on foot is not considered).

The model also assumes that there is sufficient supply of health products at the distribution warehouse to meet the demand requested by health facilities. If supply is limited, the user must adjust the requested demand in the input file. Therefore, a constraint in RoOT is that the total demand is met. An accompanying constraint in RoOT is that each health facility is visited exactly once by one vehicle. 

RoOT will schedule as many routes as needed to fulfill the distribution. If only one vehicle is available, that vehicle may be assigned several routes, each route being completed within the limit set by the user. If more vehicles are available, RoOT assigns the number of routes to available vehicles as evenly as possible. For example, if two vehicles are available, a solution may assign two routes to one vehicle and one route to a second vehicle.

RoOT assumes that the health facilities can properly store the quantity of health products requested. However, if the quantity requested by the facility exceeds its storage capacity, the model does not use this as a constraint. Instead, RoOT provides a warning to the user that the demand exceeds storage capacity, but it is still possible to execute the optimization model.

The constraints in the optimization model also include vehicle capacities for cold and ambient health products. Several types of vehicles are allowed, such as trucks, jeep, and motorcycles. For example, a motorcycle may be able to transport a small vaccine carrier with specific storage capacity for products requiring cold storage and have some space to carry other health products, like syringes or essential medicines. On the other hand, a refrigerated truck may be able to carry a larger quantity of products requiring cold storage and have capacity for other health products as well.

The last assumption is that the vehicles can travel on roads. To include distribution to an island that requires transport by boat, we selected a location that is accessible by land vehicles, and assumed that a boat would meet the vehicle at that location. A similar adjustment can be made to meet vehicles on roads if foot access is required. The constraints in the optimization model ensure that routes use roads that are accessible. A road that is in poor condition, but passable, is allowed and a penalty for the road condition is added into the risk objective function.

### Usability

**Figure 2.**
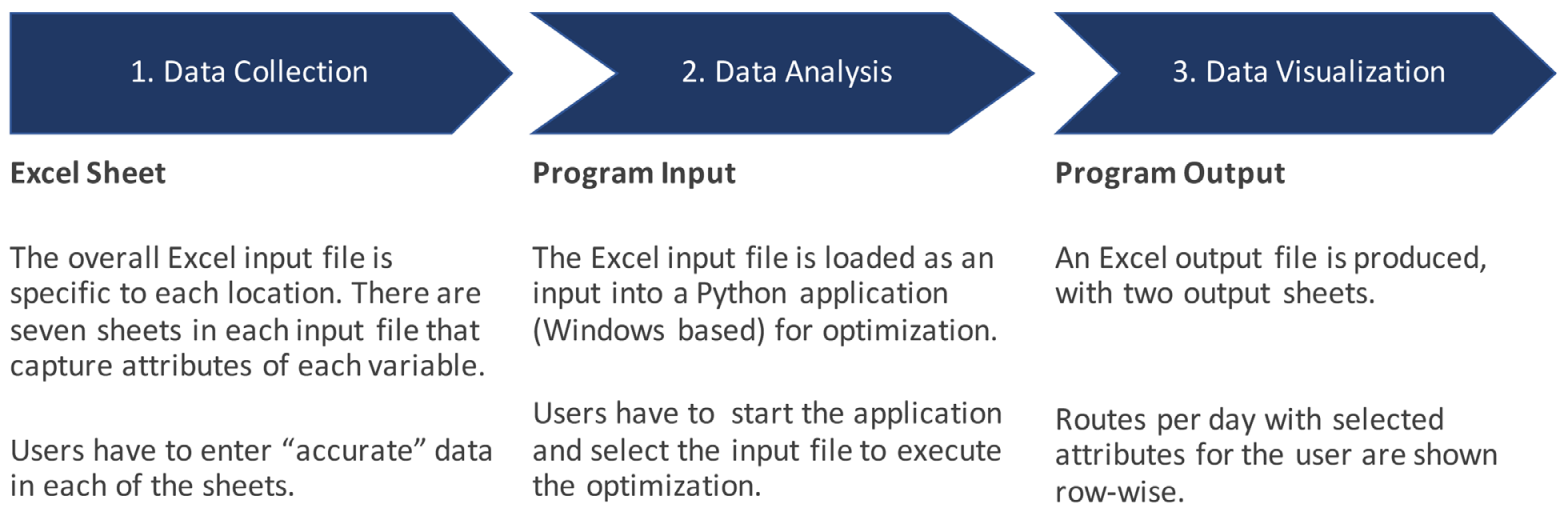
Workflow for using RoOT.

User centered design and ease-of-use was important in the tool from the very initial stages. There are several optimization and modeling supply chain tools available but, due to the design and complex interface, they are not easily used or adopted by government logisticians or technical partners that support governments. To focus on user centered design, we first mapped out the workflow for using the tool as shown in Figure 2, and analyzed the usability by using a modified version of Nielsen's Usability Heuristics ^26^, which are an industry standard in usability studies.

Based on the workflow and principles of information architecture, three principles for improving the usability of the tool were identified: (1) reduce input data errors, (2) immediate feedback to users, and (3) reduce data input time.

To reduce user errors during data input, we used dropdown menus wherever possible for the user to select options from a list rather than typing them out or copying from another sheet, based on Nielsen’s 1 ^st^ heuristic that users should get immediate feedback to make informed decisions, and 5 ^th^ heuristic that a system should be designed to prevent any errors. Hence, the RoOT Excel input file has dropdown menus for:

- selecting the start and return location for distribution,
- indicating whether a health product requires cold storage,
- vehicle availability and vehicle condition, and
- road condition.

A common challenge with any tool using multiple databases is ensuring that facility names, products, and other input information are spelled consistently throughout so that the back-end algorithm can associate them. However, often facilities have multiple variations in spelling with slight changes across different databases. It is also tedious for the user to keep track of and correct spellings across multiple sheets or databases. To address this and make the data input process easier, the users enter the names of all facilities only once, in the “center_capacities” sheet, and the names are automatically replicated across all other sheets, reducing the chances of errors. Similarly, the names of health products are entered only once and automatically replicated across relevant sheets.

In addition to RoOT’s user guide, we added brief instructions on every input sheet for easy reference by the user and to make data entry easier ^25^. We also color-coded the cells to clearly indicate where the user needed to enter data, and where it was automatically calculated for pre-processing.

Further, to reduce the cognitive load on users when modifying the data, such as removing a vehicle from being considered for routing or dropping a specific health product, instead of relying on the user to manually copy or delete data, we included a yes/no dropdown for products and available/not available for vehicles to indicate whether the model should include them in the analysis based on Nielsen’s 2 ^nd^ and 6 ^th^ heuristic ^26^. This allows the user to enter data using words and phrases that they are familiar with, making data entry easy and reducing the chances of errors.

### Computational performance

In operations research, it is well-established that the computation time to solve a vehicle routing problem, as in RoOT, increases significantly with the number of facilities to visit, as well as the number of available vehicles ^24^. However, getting quick results within a few minutes was an essential requirement by users and, hence, we developed our own optimization algorithm for RoOT that is a variant of a Vehicle Routing and Scheduling Algorithm (VeRSA) ^27^.

VeRSA embeds an indexing rule in a branch-and-bound framework to quickly construct a feasible solution in seconds, instead of hours. The routes that are determined in one or two minutes perform nearly as well as the optimal solutions that may take hours to determine. For RoOT, we adapted the indexing algorithm used in VeRSA for health product and vaccine distribution specifically. We also embedded specific constraints directly into the feasibility check in VeRSA to speed up computation. This version of VeRSA is coded in Python, and reads and writes Excel spreadsheets. Hence, the Python code is invisible to the user, making RoOT easy to use while providing timely results for logisticians.

In earlier versions of VeRSA, the number of products also increased computation time. However, in the final version of VeRSA used in RoOT, the computation was streamlined by aggregating the products into two categories: those requiring cold storage (such as vaccines) and those kept at ambient temperatures. The Python code disaggregates the two categories of products into specific names and quantities of products in the Excel output file. Thus, there is no limit to the number of products that can be input in RoOT as long as it fits in the two categories and it does not impact computation time in the optimization. Hence, RoOT can be used to plan routing for integrated health supply chains that deliver vaccines and other health products, such as family planning, malaria etc., together. RoOT can also be used in routing vaccines for campaign and routine immunization together, instead of distributing through parallel supply chains.

Computation time in modeling and routing software is an important factor for users. Commercial software can often take hours or days to obtain an optimal solution. However, government logisticians or technical partners supporting governments, often cannot run modeling problems for so long and often require quick solutions they can use. To understand the impact of number of health facilities on RoOT’s computation time, we compared it with other commercially available software. The results showed that RoOT performed very well ^21^. For 10–20 facilities, the performance of RoOT’s indexing method was similar to the best of the commercial software packages and produced an optimal solution within 2 minutes. Hence, the default computation time in RoOT is set to 2 minutes. Additionally, we also tested RoOT for greater number of health facilities to reflect distributions in larger countries. For 50 health facilities, RoOT performed better than the commercial software, providing good results in 2 minutes while the commercial software took much longer ^21^. For 100 facilities, RoOT determined a feasible solution within 2 minutes; however, this solution did not perform as well as a solution found after running a commercial software package for several hours. An advanced user can increase the default computation time of 2 minutes in the RoOT Excel input file, and in theory, RoOT will eventually obtain an optimal solution similar to commercial softwares. For practical purposes, we recommend using RoOT with 50 facilities or less to obtain optimal results in 2 minutes.

The number of vehicles used in the model also impacts computation time. To keep the computation time low keeping the user needs in mind, we recommend limiting the number of available vehicles to five or less. Based on our experience, we believe that five is a reasonable number as most provinces and districts in low- and middle-income countries often do not have more than five vehicles (with accompanying personnel) available to implement simultaneous routes. However, in a situation where more than five vehicles are available for distribution, it is possible to increase the number to more than five with the understanding that the computation time should be increased from the default of 2 minutes to provide a good solution.

The output generated by RoOT is similar to that of commercial solvers for relatively small datasets (10 or less health facilities), and RoOT provides a feasible solution faster than commercial solvers for large datasets (50 health facilities) based on a numerical comparison ^21^. This confirms that RoOT gives good results in a timely manner that are correct and represents the information provided in the input files. It is also important to emphasize that unlike commercial solvers and most of the routing tools in the market, RoOT is open-source and freely available on GitHub (see Software availability section for details), in addition to being easy-to-use ^25^.

### Operation

RoOT is an open-source tool available online for free. The current tool runs on a Windows computer, 64-bit, with Microsoft Excel version 2007 or later. There are no specific RAM requirements, but the RoOT folder needs about 1.1 GB of memory. To check if your computer is 64-bit, go to “Display Settings” and scroll down to find “About” on the left menu. When you click on “About,” you can see: “System type: 64-bit operating system.”

## Challenges and limitations

As with any modeling tool or software, the outputs are only as good as the input data. In many situations, accurate data may not be available and users have to fill data gaps using proxy data or by making assumptions. Some of the challenges and limitations of RoOT are outlined below.

***Troubleshooting when RoOT does not run:*** RoOT is a light-touch Excel-based tool that is easy to run. However, if the data in the input file is incomplete, the model does not run or provide results. If this occurs, RoOT does not display any error message indicating to the user to check the input files for errors nor does it highlight what the error would be. We recommend that the user check each of the seven input sheets to make sure the data entered is correct and that no field is left blank. However, a challenge is that the burden of troubleshooting is put on the user.

***Storage capacity of facilities:*** Currently RoOT does not limit distribution to facilities even if the demand exceeds the storage capacity available. This could be viewed as a challenge as the model is allowing distribution even when there are storage constraints. However, in practice, many facilities find ways to store health products beyond the officially designated space for storage, e.g. dry commodities are stored on top of cupboards or in corridors. To mitigate this challenge, we created a “warning” signal that provides users with real-time feedback on storage utilization based on the quantity of health products requested by a facility. This gives logisticians and supervisors insights about storage utilization, and if it exceeds capacity they could discuss with the facility, but the optimization can still be executed.

***Using available vehicles: *** RoOT assigns routes to available vehicles, as evenly as possible, even though one vehicle may be more reliable than another one. For example, if two vehicles are available, and one is in good condition while the other is in poor condition, RoOT may assign two routes to the reliable vehicle and one route to the vehicle in poor condition, accomplishing the distribution in two days. If the user wants to explore the option of only using the vehicle in good condition, then the second vehicle should be selected as “unavailable” in the input sheet.

***Multi-day routing:*** In practice, distributions of health products from provinces and districts to health facilities often take multiple days. However, the current model only allows for trips that can be completed within 24 hours. This limits the practical use of RoOT for distributions in areas which require multi-day routes, especially health facilities that are very far from the distribution warehouse and would take multiple days to reach. However, since the distribution can be done over 24 hours, the user can consider it as 3 days with 8 hours each. To expand RoOT capabilities further for multi-day routing, certain additional factors need to be considered such as overnight accommodation locations and maintaining cold chain overnight. These should be considered when expanding RoOT to allow multi-day trips.

***Scalability to more health facilities:*** Currently RoOT is recommended for use for distributions to 50 facilities or less from a single distribution warehouse. This is a reasonable limitation, because distribution is often organized by administrative or distribution boundaries. If there are more than 50 facilities for distribution, the computation time can either be increased or facilities categorized by geographical proximity.

**Figure 3.**
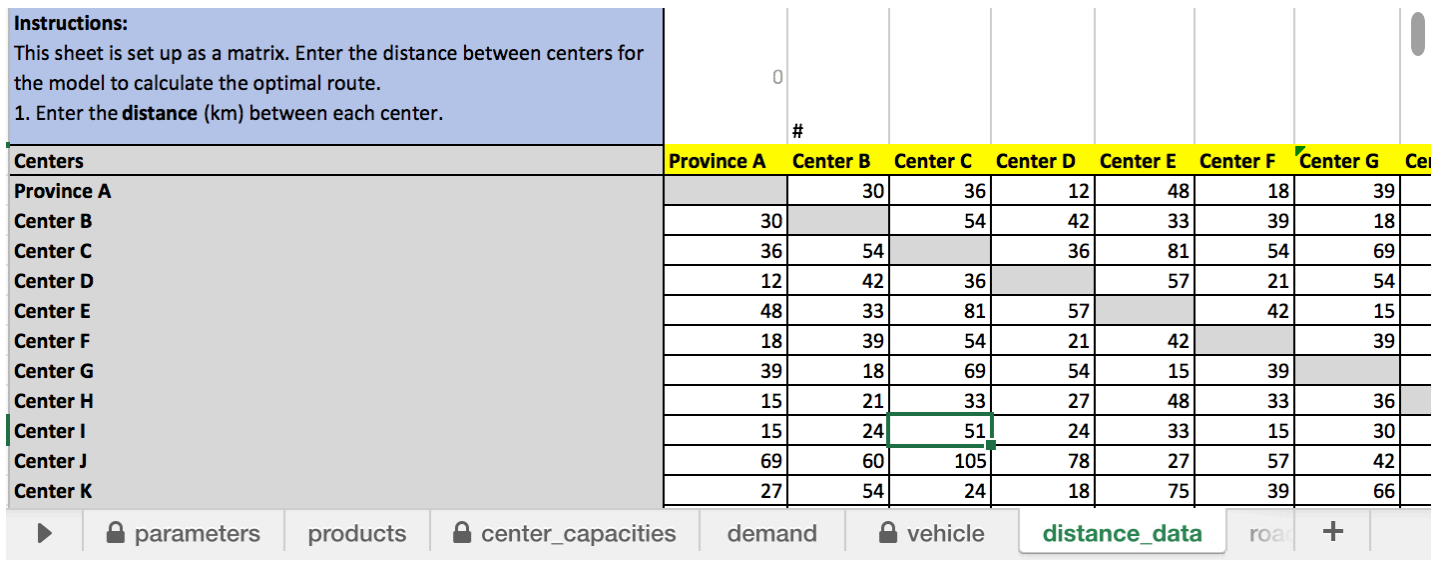
Distance matrix to fill in “distance_data” input sheet.

***Matrix of distance between health facilities:*** One of the more challenging data to input for RoOT is completing a distance matrix as shown in Figure 3. The user needs to fill all the data, and cannot leave any cell empty in order to run the model.

This requires the user to look up and enter distances between each facility, and will be even more difficult as the number of health facilities increases. Mapping software, like Open Street Maps or Google maps, may be helpful in automatically creating a distance matrix as well in identifying shared roads, such as a highway or major arterial. However, health facilities in most LMICs are not easily located in mapping software, because either they are not marked or their names may be spelled differently making it challenging to use mapping software. This could be mitigated if geo-coordinates were available for health facilities making it easy to locate on mapping software; unfortunately, those are also not readily available. In system design studies using more sophisticated modeling software, we often have to search and lookup each health facility; and in circumstances when that is not available we often approximate to the district or province’s location. While this continues to be a challenge, the user needs to only set up the distance matrix once and logisticians can also estimate the distance based on local knowledge.

***Visualization of outputs:*** One of the requirements by users was the ability to visualize the outputs and see the routes on a map, but RoOT is currently limited to generating results in an Excel output file with no visualization. As mentioned above, it is challenging to integrate mapping software (e.g., Open Street Maps or Google maps) with RoOT, but is a possibility for future work.

## Use cases

### Training logisticians in Mozambique

RoOT is designed to meet the modeling needs of government stakeholders and users, including time available, resources, technology, ease of use and capabilities. To understand if our tool meets the requirements of the user, we trained eight logisticians in Mozambique representing provincial and district levels. We designed a four-step assessment to measure and understand how government users received the tool, based on the following questions:

1. *Reaction: Do you like the tool and is it easy to use?* This was measured through observation and survey.
2. *Learning: Do trainees leave the training understanding how to use the tool?* This was measured through a practical exercise and survey.
3. *Behavior: Do logisticians actually use the tool to plan distributions?*
4. *Results: Does using the tool lead to better outcomes?*

The participants of the training described that currently they follow pre-determined routes, starting either with the closest health facility or the largest one. Often, they find out which vehicle is available for distribution at the last minute when it arrives at the distribution facility, and they must quickly adjust the routes accordingly. The participants agreed that the tool was easy-to-use and would help them in distributions as illustrated from these quotes:

“ *We were creating the routes in an ad-hoc way. We didn’t have a platform to guide us to calculate the routes and the quantities per route. This tool can help us by giving us different ways of arriving at the health facility.*”
“ *The truth is that we were working in the dark, we first tried something on the ground, then we would know the estimated cost and time, and whether that works or not; but with the tool, we’re not in the dark. Calculating the time used in the distribution is one of the hardest things to do, as sometimes we don’t know how to calculate whether we’ll be able to return on the same day or the next; and this helps us calculate the time. But it should still consider the fact that you sometimes have to come back the next day, not always on the same day.*”
“… *the tool tells us what is the capacity at each health facility also helps us to visualize. We used to load vaccines according to needs only and not take into account what is the actual capacity at the health facility.”*

Over 60% of the logisticians in the training rated their confidence to use the tool as skillful, i.e., they could use the tool independently with occasional help from a specialist. Out of the eight participants, seven were confident in being able to use the tool to determine routes and to decide which vehicles to use. The logisticians confirmed that they would be able to use the Portuguese version of the tool for routine and emergency distribution. Unfortunately, due to the COVID-19 pandemic in 2020, the full deployment of RoOT has been delayed.

### Using RoOT for distributions during an emergency

RoOT is designed to meet the needs of government stakeholders for routine as well as emergency distributions. For routine distribution, we anticipate that logisticians would use the tool to determine a number of consistently-used routes, updated with current road and vehicle conditions.

RoOT can also be used for emergency situations, like outbreaks or campaigns when a new health product needs to reach health facilities quickly. As supplies and treatments for COVID-19 become available or a new vaccine is introduced, governments will need to mobilize quickly to make sure the vaccine and health products are getting to the most vulnerable people as soon as possible. Even though manufacturers are trying to build capacity for a new vaccine, there will be supply shortages as all countries strive to procure it. Hence, countries will need to prioritize how many vaccines to deliver and to which health facilities in the fastest way possible, while reducing the risk to potency. RoOT can be used to quickly determine and plan routes based on minimizing transit time and risk, to align with government priorities.

To illustrate the use of RoOT in an emergency situation, we consider a natural disaster, such as a cyclone. In an emergency situation, there are several parameters that a logistician may need to assess and modify. In this cyclone scenario, distributions need to take place from a different warehouse than what is typically used. In addition, several health facilities cannot accept supplies because their storage capability has been destroyed, and several roads are not accessible anymore.

**Figure 4.**
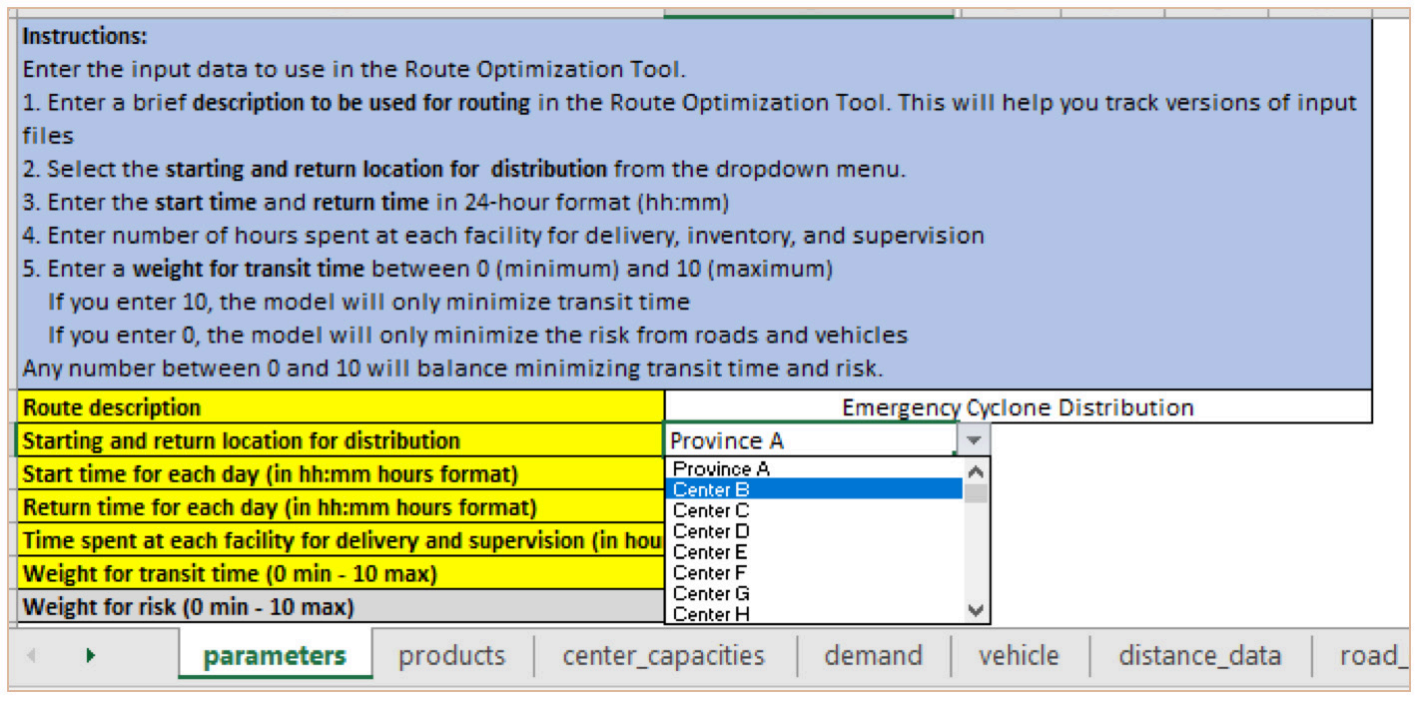
Change the distribution warehouse location from Province A to Center B in the “parameters” input sheet.

***Step 1: Changing distribution warehouse location***. The user would change the start and return location for distribution in the “parameters” input sheet by selecting another facility from the dropdown menu. As shown in Figure 4, the distribution location has changed from Province A to Center B.

**Figure 5.**
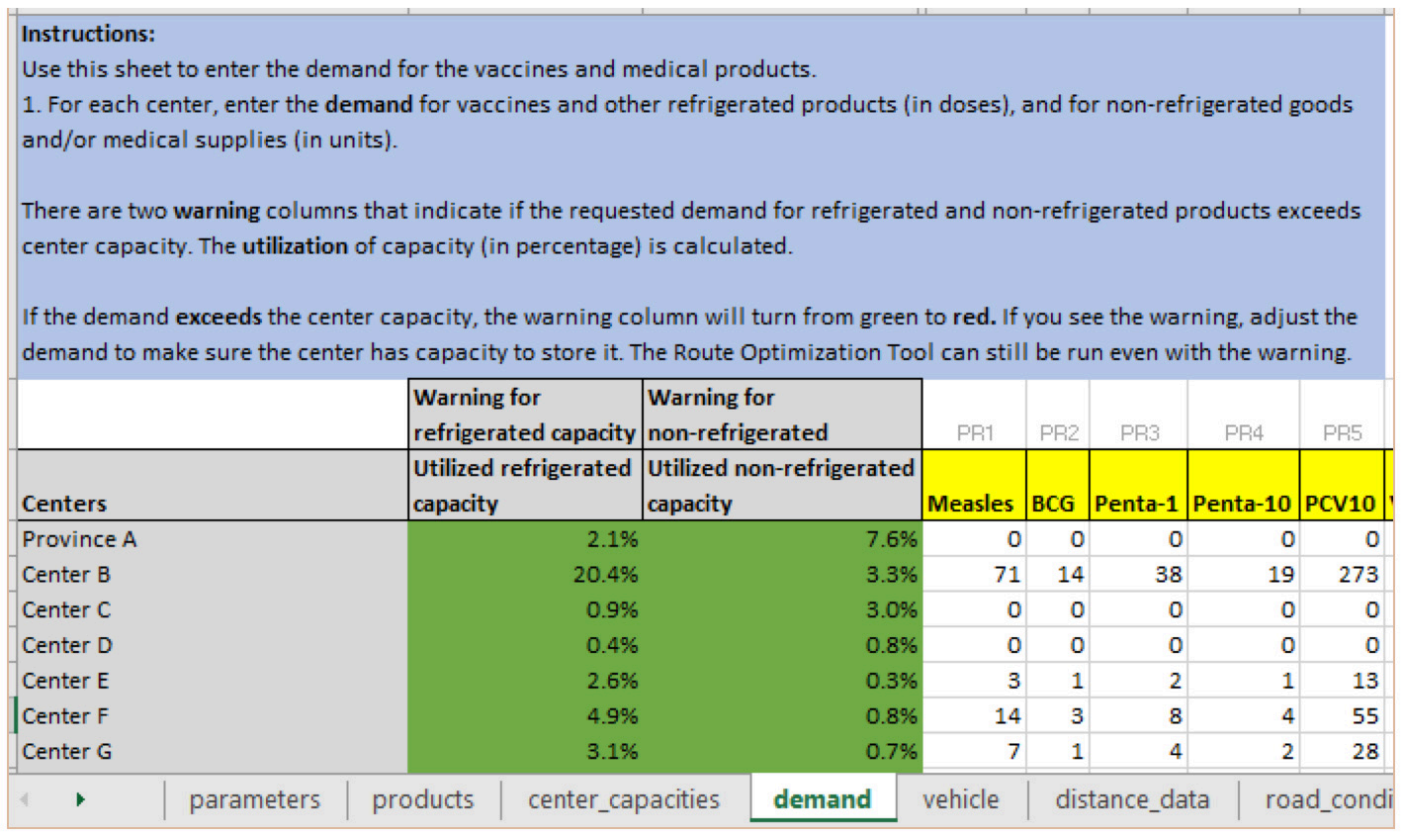
Change the demand for health facilities; set demand to zero for facilities that are not able to store health products at this time and adjust demand for other facilities.

***Step 2: Updating demand for health facilities***. Given the emergency situation, not all health facilities are intact or have storage for supplies. Hence, health products need to be distributed to a smaller number of health facilities that may see an upsurge in demand as people from nearby areas are also traveling there to seek care. The logistician does this in RoOT by changing the demand to zero for health facilities that are not able to store supplies at this time, and accordingly increases demand for other facilities. As shown in Figure 5, Province A, Center C and Center D have zero demand, and demand has increased for Center B and Center F.

**Figure 6.**
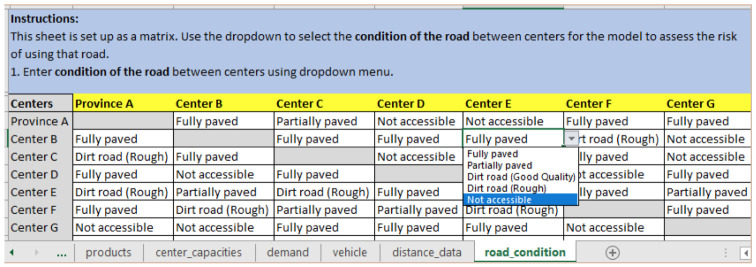
Update the “road_condition” input sheet to reflect current road conditions and accessibility between Centers.

***Step 3: Updating road conditions***. In case of a natural disaster, like a cyclone, many of the road conditions may change or become completely inaccessible due to flooding or destruction, making them unavailable for use. The logistician planning the routes can select the updated road conditions from a list of options in a dropdown menu, as seen in Figure 6, where the road from Center B to Center E is not accessible. The dropdown menu allows the selection from the following options: Fully paved, Partially paved, Dirt road (Good Quality), Dirt road (Rough), and Not accessible.

**Figure 7.**
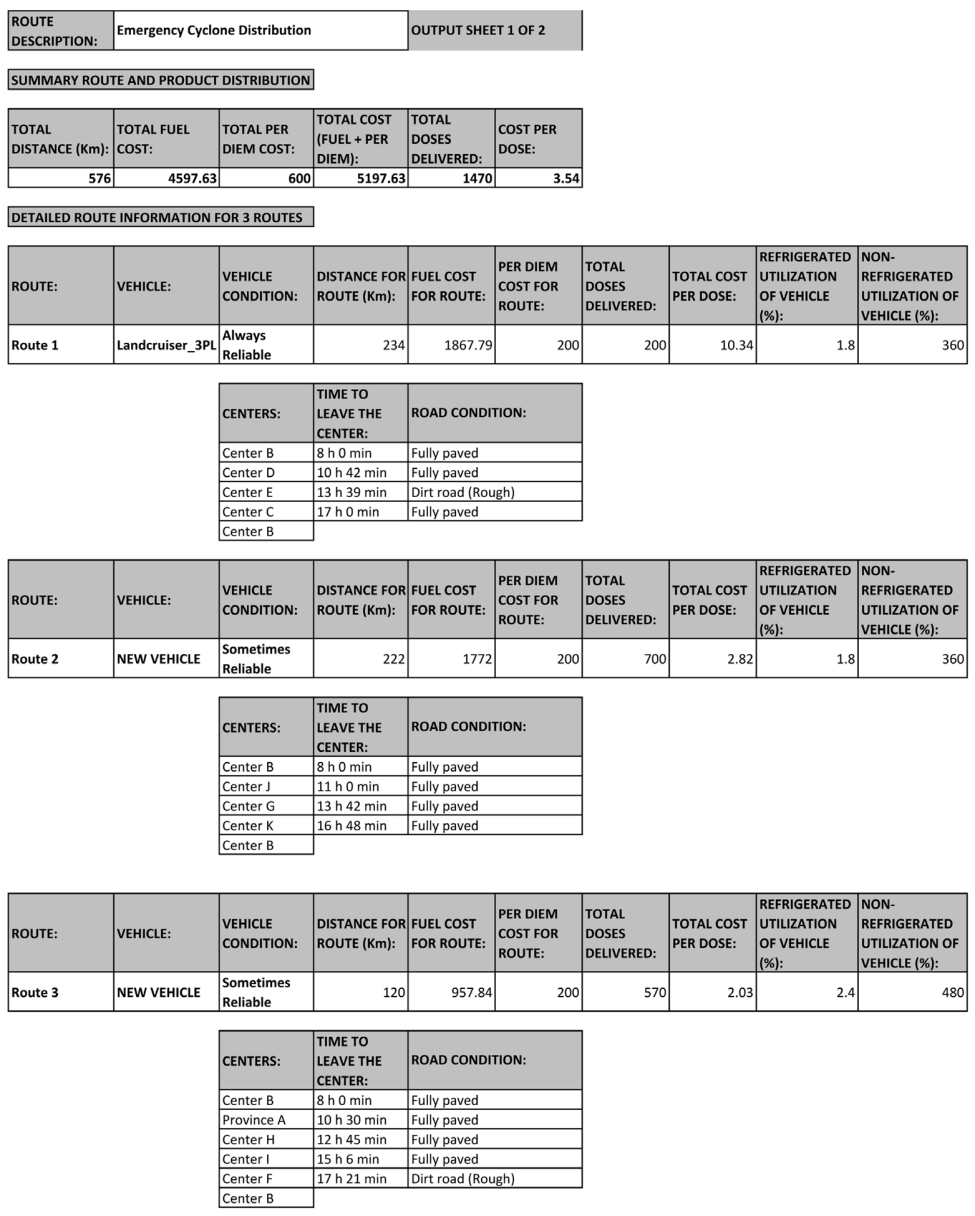
New routes for emergency cyclone distribution with two available vehicles.

***Step 4: Run RoOT for updated results***. After making all the changes to the inputs, the user should save the input file and re-run RoOT. The tool will provide results within 2 minutes, and generate an Excel output file displaying the routes, departure times, and health products that need to be delivered for the emergency situation. Figure 7 illustrates the new routes for the cyclone scenario. There are three routes that start and end at Center B. There are two vehicles available for distribution, the Landcruiser_3PL, and a New Vehicle. As shown in Figure 7, the Landcruiser_3PL leaves Center B at 8am, and visits Centers D, E, and C before returning to Center B. The New Vehicle has two routes, as illustrated in Figure 7. Notice that these three routes never use any roads that are marked “Not Accessible” using the dropdown menu in the input sheet in Figure 6.

## Conclusions

In conclusion, RoOT is an easy-to-use optimization tool that enables logisticians to quickly plan and adjust routes for health product distribution accounting for transit time and risk of temperature excursion of sensitive products, such as vaccines. RoOT is designed to

- meet the requirements of government stakeholders, and
- provide faster results than commercial software

As users gain experience with RoOT, they will identify several areas for future improvements. One possibility is to integrate RoOT with existing software tools (such as demand projections and cost analyses) to increase consistency of data inputs. At the same time, it is desirable to maintain independent use of RoOT so it is easily transferable to many countries and many types of health products. Another future extension is to consider multi-day routes, where many considerations must be discussed and appropriate assumptions and constraints developed. Lastly, inclusion of visualization with mapping software will greatly improve the usability of RoOT.

## Software availability

Route Optimization Tool (RoOT), with user guide and underlying data is available from: https://github.com/villagereach/RoOT (in English) and https://github.com/villagereach/RoOT-portugues (in Portuguese).

Archived source code available from: http://doi.org/10.5281/zenodo.4477538 ^28^

License: GNU General Public License
