# Supplementary material for: Route Optimization Tool (RoOT) for distribution of vaccines and health products
Source: Gates Open Res. 2021 Nov 15;5:34. Originally published 2021 Mar 1. [Version 2] doi: 10.12688/gatesopenres.13219.2 (PMC8771994; doi:10.12688/gatesopenres.13219.2)
Supplement: Peer review history

# Peer review: Reviewer response for version 2

**DOI:** 10.21956/gatesopenres.14695.r31411

**Published:** 2022-01-19

**Authors:** Andrew Inglis

**Affiliations:** 1 USAID Global Health Supply Chain Programme, Procurement and Supply Management, International Business Machines, Arlington, VA, USA

Approved.

Are the conclusions about the tool and its performance adequately supported by the findings presented in the article?

Partly

Is the rationale for developing the new software tool clearly explained?

Yes

Is the description of the software tool technically sound?

Yes

Are sufficient details of the code, methods and analysis (if applicable) provided to allow replication of the software development and its use by others?

Yes

Is sufficient information provided to allow interpretation of the expected output datasets and any results generated using the tool?

Partly

Reviewer Expertise:

NA

I confirm that I have read this submission and believe that I have an appropriate level of expertise to confirm that it is of an acceptable scientific standard.

# Peer review: Reviewer response for version 1

**DOI:** 10.21956/gatesopenres.14437.r30466

**Published:** 2021-04-06

**Authors:** Andrew Inglis

**Affiliations:** 1 USAID Global Health Supply Chain Programme, Procurement and Supply Management, International Business Machines, Arlington, VA, USA

Firstly, I believe the authors have developed and demonstrated an innovative approach to solving a routing problem for distribution in a development context.

 My main reservations relate to questions 4 and 5:

- Is sufficient information provided to allow interpretation of the expected output datasets and any results generated using the tool?
- Are the conclusions about the tool and its performance adequately supported by the findings presented in the article?

The comparison against other routing options seams to be focused on other commercial options saying “Most of the routing software used in commercial logistics focuses on distribution efficiency to minimize cost and time” - no mention or consideration for other low cost / open source options that are currently available, e.g. https://www.graphhopper.com/ or https://openrouteservice.org/. It would be better for the reader to know that there are other options.

 I reviewed the reference (21 - Petroianu LPG, Zabinsky ZB, Zameer M, et al.: A light‐touch routing optimization tool (RoOT) for vaccine and medical supply distribution in Mozambique. Intl Trans in Op Res. 2020) - provided to support the following statement ”To understand the impact of number of health facilities on RoOT’s computation time, we compared it with other commercially available software. The results showed that RoOT performed very well ^21^”.

 I did not find a table or data that showed computational time between Commercial software and RoOT. I did find against different solvers - Gurobi, CBC and GLPK - how that is not the same as comparison to commercial software. The paper then indicates it did analysis against commercial software: “For 10–20 facilities, the performance of RoOT’s indexing method was similar to the best of the commercial software packages and produced an optimal solution within 2 minutes. Hence, the default computation time in RoOT is set to 2 minutes. Additionally, we also tested RoOT for greater number of health facilities to reflect distributions in larger countries. For 50 health facilities, RoOT performed better than the commercial software, providing good results in 2 minutes while the commercial software took much longer ^21^ . For 100 facilities, RoOT determined a feasible solution within 2 minutes; however, this solution did not perform as well as a solution found after running a commercial software package for several hours.”

 Therefore, since they did this analysis it would be good to include this data within the paper for the reader to see and understand the differences.  Additionally, when the default computation time in RoOT is set to 2 minutes, the same setting for computation time could be set for commercial software and then comparison would be on how well the solutions were after 2 mins determined between RoOT and the commercial software.  Thus, we don’t know if the solution calculated after 2 mins was similar or not between RoOT or the Commercial Software; having this information this would strengthen the evidence.

 “Computation time in modeling and routing software is an important factor for users. Commercial software can often take hours or days to obtain an optimal solution.” Further context to this statement would be useful to the reader, if you are looking to solve a large routing problem to fully optimal solution then it will take time if using the RoOT or Commerical Software or other open source routing software. Just saying “Commercial software can often take hours or days” without context is misleading. This is then shown when context is then provided in the paper. The paper states for a small problem “For 10–20 facilities, the performance of RoOT’s indexing method was similar to the best of the commercial software packages and produced an optimal solution within 2 minutes” thus they take the same time. Then for a slightly larger problem “For 50 health facilities, RoOT performed better than the commercial software, providing good results in 2 minutes while the commercial software took much longer ^21^ .” This is the “sweet spot” for this tool and thus should emphasized as in the environment this tool is to be deployed in there are a number of distribution points that could meet this criterion. Then for a larger problem  “For 100 facilities, RoOT determined a feasible solution within 2 minutes; however, this solution did not perform as well as a solution found after running a commercial software package for several hours”, this where you are starting to step out of the “sweet spot”, however as described in the paper for practical purposes it is good enough. Thus, the importance for having that table to demonstrate the difference in computation time.

 On the Distance Matrix the paper does recognize the challenge in filling this out. Suggestion is to mention such resources to support the distance matrix https://openrouteservice.org/ that provides distance matrix, this could be useful as it is designed to work in this context.

 Additionally, “Updating road conditions” is a simplification of the impact and ease of determining which paths between different sites have been affected by a disaster or change in the road conditions. A change in road condition is unlikely to just affect one pair of sites as described in the paper, it would need to be determined if and how affect all paths to a site across matrix. For example if a road is inaccessible “from Center B to Center E is not accessible” it could affect the paths between all sites to B and/or all sites to E.  As it is unlikely to have a single path that is only between sites B and E, it is more common that sites will share a road with other paths to sites, thus when a road is affected then it will be across multiple sites. Thus, it would be good to clarify and expand the current clarification on how to update the road conditions and ensure that readers understand what needs to be done when inputting updates to road conditions.

Are the conclusions about the tool and its performance adequately supported by the findings presented in the article?

Partly

Is the rationale for developing the new software tool clearly explained?

Yes

Is the description of the software tool technically sound?

Yes

Are sufficient details of the code, methods and analysis (if applicable) provided to allow replication of the software development and its use by others?

Yes

Is sufficient information provided to allow interpretation of the expected output datasets and any results generated using the tool?

Partly

Reviewer Expertise:

NA

I confirm that I have read this submission and believe that I have an appropriate level of expertise to confirm that it is of an acceptable scientific standard, however I have significant reservations, as outlined above.

# Response

**Published:** 2021-10-26

**Authors:** Mariam Zameer

1. We have added Graphhopper and Openrouteservice to the paper. Thank you for highlighting this. 
2. The reviewer makes a good point to distinguish “commercial software” with “solvers”.  We have clarified this in the text.
3. Thank you for this suggestion to fill out the Distance Matrix. We added, “Mapping software, like Open Street Maps, Google maps, or https://openrouteservice.org/ …”
4. We added on updating road conditions, “If a road is blocked due an emergency or some other reason, the user updated it on the “road_condition” input sheet (Figure 6) to ‘Not accessible’. RoOT will not use this road while creating a route, and determine alternative routes. If the blocked road is used by several facilities, it must also be changed to ‘Not accessible’ in the distance matrix.  If a facility is completely inaccessible by existing roads, a make-shift facility can be created to the point which is accessible by road.”
  We would be glad and eager to work with openrouteservice in the future, to improve this.

# Peer review: Reviewer response for version 1

**DOI:** 10.21956/gatesopenres.14437.r30465

**Published:** 2021-03-24

**Authors:** Ryan McWhorter

**Affiliations:** 1 UNICEF, Copenhagen, Denmark

I enjoyed reading the article and see value in the tool. I look forward to using it in the field.

 Please see my comments below for the authors' consideration.

**Abstract:**

- I think the first sentence’s sentiment is borrowed from an oft-cited statement that immunization is one of the most cost-effective interventions for children’s health. Instead, this statement says that “Delivery…is one of the most effective interventions to ensure availability of supplies…”. I think it’s obvious that the delivery of a product is one of the most effective interventions to having the product available. I don’t think I’d call it an intervention, though. Delivery is just part of the operation, not an intervention. I recommend the authors consider a different intro statement regarding the importance of delivery.

**Introduction:**

- The first paragraph focuses on the efficient and optimized distribution. While budgets are a concern for COVID response, I think these opinions deserve a reference. Are governments more concerned with effective distribution or efficient distribution? It could be (and likely is) both, in which case this paragraph would suggest that both areas need to be addressed in the planning and in the tool. I think the paper goes on to support this idea by having both risk and speed in the objective function, but this sentence tends to focus only on efficiency.
- The third paragraph warrants a reference for both “one of the most important decisions” and for comments about “no tools”, even if this is clarified later in the article, it could reference these points.
- The fourth paragraph warrants a reference for impacts of Idai on Mozambique’s health supply chain.

**Methods:**

- Box 1: is there an article/report/survey/feedback to reference for these questions?
- Constraints: Is there a need for a constraint on the “driver’s day”? For example, that the driver can spend only X hours on the clock?
- For distances, the time factor may be a more relatable quantification than distance, especially if taking into account transit through urban areas and roads that are known to be in poor condition.

**Usability:**

- In the comment regarding existing tools in the first paragraph and their usability, suggest a reference for the statement or reference back to Landscape Analysis.
- A table or graphic representing the Nielson heuristics and recognition in the tool would be helpful, as the reader cannot keep track of the 1 ^st^, 5 ^th^, or 6 ^th^ referenced heuristic.

**Computational Performance**

- Are the two product categories limited to cold chain and ambient, and is cold chain assumed to be 2-8C? This could be better defined in the text for the reader.
- This is not my area of expertise, but I imagine that software developers would want to see more data in this section as it pertains to comparisons and the definition of “good results” as it applies to testing against 50 facilities. I listed in my answers above "Partial" on this section because I don't know as much as software developers regarding this section in terms of ability to replicate or analysis.
- Is there a reference (report, survey, publication) for the “reasonable number of vehicles”? I would agree with the statement, but I don’t have proof.

**Use Cases:**

- Training: I didn’t see answers to all four posed questions? It looks as if only reaction and learning are answered…what about behavior and results?

**General Comments**

- It would be helpful for the reader to outline the scope of the tool at the top of the article. As I understand, it’s for optimizing routing from 1 location to N locations – so the focus is on the location that manages distribution downstream (as opposed to say, an area that has multiple distribution points or a facility that can both drop-off and pick-up goods).
- Recommend to the authors to run the text by a copy editor to clean up grammar and word choice.

Are the conclusions about the tool and its performance adequately supported by the findings presented in the article?

Partly

Is the rationale for developing the new software tool clearly explained?

Yes

Is the description of the software tool technically sound?

Yes

Are sufficient details of the code, methods and analysis (if applicable) provided to allow replication of the software development and its use by others?

Partly

Is sufficient information provided to allow interpretation of the expected output datasets and any results generated using the tool?

Yes

Reviewer Expertise:

Global health and immunization supply chain planning

I confirm that I have read this submission and believe that I have an appropriate level of expertise to confirm that it is of an acceptable scientific standard, however I have significant reservations, as outlined above.

# Response

**Published:** 2021-10-26

**Authors:** Mariam Zameer

**Abstract** : This is a good point, and we have modified the introductory sentence.

**Introduction:**

- We modified the sentence to include efficient and effective, “This is even more critical today with the COVID-19 pandemic, where efficient and effective distribution of the COVID-19 vaccine is needed.”
-  Reference added for “one of the most important decision”. Sentence changed about “tools” as the landscape and literature review is summarized in the paper under “Landscaping of existing methods and tool”.
- Reference added on impact of Idai.

**Methods:**

- VillageReach is a close supply chain partner with the government in Mozambique, and the questions came from several discussions and meetings with government stakeholders.
- Yes, there is a need for a constraint on the length of a “driver’s day”. This can be reflected in the start time and return time of the parameters sheet (figure 1). A sentence has been added to clarify this.
- Yes, the time factor could be more relatable. However, it might change during the time of the day, especially in urban areas. We decided to use distances between facilities and allow for different vehicles with different speeds, so in this way, we do consider time but assume it is the same throughout the day.

**Usability:**

- Thank you for the suggestion. We added a reference back to the section *Landscaping of existing methods and tools*.
- Thank you for the suggestion. A table summarizing the usability heuristics has been added, and language simplified for easier reading. 

**Computational Performance:**

- We only consider two product categories, cold chain and ambient.  We do not assume a temperature range for the cold chain, or for ambient temperature. When the user identifies the vehicles with cold storage, they can determine an appropriate temperature range. We reworded this in the text as follows, “… aggregating the products into two categories: those requiring cold storage (such as vaccines) and those kept at ambient temperatures. We do not assume a temperature range for the cold chain, or for ambient temperature. When the user identifies the vehicles with cold storage, they can determine an appropriate temperature range that satisfies the health products’ requirements (e.g., 2-8 ^o^C for many routine vaccines).”
- This was also requested by another reviewer. We expanded the paragraph on computation time and referred to Petroianu *et al*. for detailed results.
- This number came from experience and discussions with stakeholders, and a phrase is added, “Based on our experience and discussions with stakeholders, …”

**Use Cases:** Unfortunately, due to the COVID-19 pandemic in 2020, the full deployment of RoOT has been delayed, and we are not able to answer all the questions.

**General Comments:**

- It can be used by any facility distributing products – whether it’s one big provincial warehouse or multiple large facilities delivery to smaller facilities. We have added a line to clarify this.
- Paper has been copy edited and cleaned for grammar and easier reading.

# Peer review: Reviewer response for version 1

**DOI:** 10.21956/gatesopenres.14437.r30463

**Published:** 2021-03-18

**Authors:** Hua Ni

**Affiliations:** 1 USAID GHSC-PSM, Arlington, VA, USA; 2 IBM GBS, Falls Church, VA, USA

The paper describes the RoOT for distribution of vaccine. This tool addresses a very practice needs of supply chain last mile distribution with dynamic routing capability. I applaud the authors’ effort to create an open source solution that factors the operational environment to make the tool more accessible for broader usage in the field of LMIC countries.

 Among the questions raised to the reviewer, I answered “Partly” for the following two questions.

1. Are sufficient details of the code, methods and analysis (if applicable) provided to allow replication of the software development and its use by others?
2. Is sufficient information provided to allow interpretation of the expected output datasets and any results generated using the tool?

Here are some specific comments that I suggest the authors consider, either providing additional details or making some clarifications:

1. Page 4: the comments around existing landscape and in particular on the final two candidates are a bit subjective in the absence of further details. I suggest providing some examples on the complexity, cost, and skills needed to operate.
2. Page 5: if vehicles have fixed ambient and cold capacity, it seems that the constraint of requiring exactly one vehicle to visit each health facility may be too limiting. It is plausible to result in lower vehicle utilization given either ambient or cold storage could become the bottleneck first.
3. Page 5: it is not clear whether the transit time refers to the whole route or just the portion that still has vaccine onboard. If we are to reduce spoilage probability, it seems to be the case that we want to minimize the transit time when vaccine is still onboard the vehicle, and the empty return segment is less critical since no vaccine is onboard, and cost doesn’t seem to be part of the stated objectives.
4. Page 5: while the weight used to combine the two objectives is easy to use, it is prone to misuse/misinterpretation. One may think 5 and 5 would put transition time and risk penalty equally important, but without knowing the scale of the individual objective values, the effect is unpredictable. I suggest providing some additional guidance on the nuances here.
5. Page 5: it is not clear as how risk penalty objective is calculated. I suggest providing some clarification.
6. Page 6: it is not clear if the vehicles are of different capacity or have the same capacity and just differ by the risk factor. Suggest providing some clarification.
7. Page 8: while it references the prior page on VeRSA, it may be beneficial to add a high-level recap of the algorithm.
8. Page 9: when a road is blocked due to emergency, it would have cascade of impacts on the OD matrix. I suggest providing some guidance to the users here.
9. Page 12: the screenshot seems to indicate that Non-Refrigerated Utilization of Vehicles at 360% which seems to be wrong. The Refrigerated Utilization of Vehicle is at 1.8%. Is it out of the total capacity of the vehicle? I suggest updating the tables.
10. Page 13: it may be beneficial to cite some solution quality result from the MZ operation to further strengthen the conclusion of the effectiveness of the tool.

As I indicated, I applaud the authors’ effort, and hope my reviews are beneficial to strengthen this already strong paper. All views expressed here are my personal views and don’t represent any of my affiliations.

Are the conclusions about the tool and its performance adequately supported by the findings presented in the article?

Yes

Is the rationale for developing the new software tool clearly explained?

Yes

Is the description of the software tool technically sound?

Yes

Are sufficient details of the code, methods and analysis (if applicable) provided to allow replication of the software development and its use by others?

Partly

Is sufficient information provided to allow interpretation of the expected output datasets and any results generated using the tool?

Partly

Reviewer Expertise:

Not in a research role. Education background in operations research, specializing in mathematical optimization. 5+ years of experience in public health supply chain operational analytics, including last-mile distribution.

I confirm that I have read this submission and believe that I have an appropriate level of expertise to confirm that it is of an acceptable scientific standard, however I have significant reservations, as outlined above.

# Response

**Published:** 2021-10-26

**Authors:** Mariam Zameer

1. We added some more details based on practical experience of using the tools: “Also, based on our experience, they require detailed data that is often not readily available in LMICs, such as geographic coordinates or details of road networks. The user interface of the existing tools is complex for those who may not be familiar with it which requires technical assistance that can be costly to some governments”

 2. Our model chooses one vehicle at a time to create a route, resulting in that vehicle visiting as many facilities as possible during its route, according to its capacity. Therefore, its utilization would be generally high. If we allow more than one vehicle to visit each center, it would result in higher transit time and lower vehicle utilization.

 3. Yes, considering minimizing the transit time only when vaccine is onboard would be an interesting future improvement, whereas the model minimizes the total transit time of all routes (updated in the text).  The risk penalty representing vaccine spoilage is considered for each segment of the total route. It is important to keep track of the total transit time, including the empty return segment, to ensure the maximum time spent on a route during a single day is respected. 

 4. That is good feedback that it is prone to misinterpretation. We normalize the objective values so the chosen weights affect them similarly. As suggested, we added an explanation; “The tool normalizes the values of each objective, so the weights affect each objective similarly. For example, weights of 5 and 5 give equal importance to transit time and risk, due to the normalization.”

 5. Thank you for the suggestion.  We added some more details, and “The user classifies the vehicle and road conditions, which the tool converts to a number and uses to calculate risk”

 6. We added, “Different types of vehicles may have different risk penalties, as well as different storage capacities.”

 We have clarified another paragraph later to indicate the user enters the transport capacity for each vehicle: “The user enters the cold and ambient transport capacity for each vehicle, and this is a constraint the model considers when optimizing routes. The user can enter any type of land vehicle and its transport capacity. For example, a motorcycle’s cold storage capacity may be the capacity of a small vaccine carrier with some additional space to carry ambient products, like syringes or essential medicines. On the other hand, a refrigerated truck would have a larger transport capacity for cold and ambient products, which the user would input.

 7. Thank you for the suggestion.  We added, “VeRSA calculates two indices based on the problem constraints to decide which vehicle to use for the route and which center to visit next. Every time that a new center is visited and added to the solution, the index is recalculated to choose the next best center to visit. Therefore, the indices are used to create good and feasible routes in a timely manner.”

 8. We added some more details in the text and updating the “road_condition” matrix if a road is blocked.

 9. Thank you for catching this. You’re correct, and we have updated figure 7.

 10. We had great feedback from the users, but did not have the opportunity to fully deploy the tool, due to the COVID pandemic. We are looking for opportunities to deploy the tool, and open to collaborations.
